# Severe Acute Respiratory Syndrome Coronavirus 2 Reinfection Cases Corroborated by Sequencing

**DOI:** 10.4269/ajtmh.21-0365

**Published:** 2021-08-09

**Authors:** Jonathan Massachi, Kevin Christopher Donohue, John Daniel Kelly

**Affiliations:** ^1^ School of Medicine, University of California, San Francisco, California;; ^2^ Department of Epidemiology and Biostatistics, University of California, San Francisco, California;; ^3^ Institute of Global Health Sciences, University of California, San Francisco, California;; ^4^ F. I. Proctor Foundation, University of California, San Francisco, California;; ^5^ San Francisco VA Medical Center, San Francisco, California

## Abstract

Evaluating cases of reinfection may offer some insight into areas for further investigation regarding durability of immune response to severe acute respiratory syndrome coronavirus 2 (SARS-CoV-2). Sixty cases of reinfection with viral sequencing were identified in PubMed, Embase, Web of Science, and medRxiv before May 1, 2021.Episodes of infection were separated by a median of 116 days. Severity of illness was greater among individuals reinfected within 90 days of initial infection, no asymptomatic initial cases developed severe reinfection, nearly half of cases had suspected escape variants, and nearly all individuals tested following reinfection were found to have detectable levels of anti-SARS-CoV-2 antibodies. This analysis is limited by the heterogeneous methods used among reports. Reinfection continues to be relatively rare. As the case rate presumably increases over time, this review will inform measurements to determine the natural history and causal determinants of reinfection in more rigorous observational cohort studies and other standardized surveillance approaches.

## INTRODUCTION

Reinfection of a small fraction of > 85 million cases would be a major public health concern, prolonging the severe acute respiratory syndrome coronavirus 2 (SARS-CoV-2) pandemic. Given the recent discovery of SARS-CoV-2, the scientific community has relied on reinfection data from endemic coronaviruses, which suggested a small but significant proportion of reinfections occur by 12 months, to inform the potential for SARS-CoV-2 reinfection. Over the months since, a global registry of SARS-CoV-2 reinfections was created and there have been a small but increasing number of case reports.

To ensure that identified cases represent true reinfection, standardized methods, such as those designed by the CDC, are of great importance to developing a clear understanding of the frequency and features of reinfection. Paired sequencing of viral samples from initial and reinfection positive tests has aided in identifying cases likely to be due to reinfection with a phylogenetically distinct strain rather than persistent shedding.^1^^,^^2^ In recent months, reports of reinfection supported by paired sequencing results have emerged internationally.^3^^–^^13^ Identification of cases of reinfection supported by sequencing results around the globe suggest that this is an appreciable entity and is likely occurring on a greater scale than is currently reported given the limitations on testing and sequencing. This has left many open questions regarding the rate of reinfection, potential infectiousness of reinfected individuals, and factors mediating the effectiveness and durability of immune response to initial exposure or infection.

Many hypotheses have begun to emerge in the literature regarding factors that may influence the risk of reinfection occurring or the potential for clinical severity of reinfection. Many of these are based on the ability of individuals to mount an adequate immune response to initial infection and the duration for which that immune response is expected to remain. Several hypotheses that are of particular interest are that escape mutations enabling evasion of established immune responses would result in a greater probability of reinfection, individuals who experienced a less severe clinical syndrome during initial infection (especially asymptomatic individuals) may be at greater risk,^3^^,^^4^ or that as neutralizing antibody titers begin to decline (by 3 months following initial infection), individuals may be at greater risk for reinfection.^5^^,^^6^ Lineages such as P.1 and B.1.1.7 that carry mutations in the spike proteins have been associated with an appreciable number of case reports of reinfection included in this case review.^7^^–^^14^ In a small sample of asymptomatically infected individuals, neutralizing antibodies and anti-S1 IgM were not detectable by 2 months following initial infection and up to 38% of asymptomatic individuals did not have detectable neutralizing antibodies at any point.^15^ Studies have indicated that neutralizing antibody titers appear to correlate with protection from infection and begin to decline as early as the first month following symptom onset, leaving concern for the level of protection that remains, whereas T cells have been shown to be maintained for at least 6 months following initial infection.^16^ Although answering these questions definitively requires a structured observational study, commonalities between reported cases of reinfection may offer some insight into additional features to investigate further.

With the development, emergency approval, and subsequent rollout of several SARS-CoV-2 vaccines globally, many questions still remain regarding the potential for subsequent infection following immunization as well as the durability of the protective effects observed in the vaccine trials. The specific answers to these questions are limited by the availability of longitudinal follow-up on trial participants that will expand in the coming months and years. Meanwhile, examining cases of reinfection among individuals who had previously experienced viral infection may offer some insight into patterns or causal determinants of reinfection that may also be relevant to vaccinated individuals or may at least help elucidate the potential benefit of vaccinating individuals who have recovered from SARS-CoV-2 infection.

To date, reports of reinfection exist as isolated case reports. The aims of this article are to summarize the findings of these reports and identify commonalities or trends that emerge and may serve as causal determinants of reinfection.

## METHODS

### Information sources and search.

The PubMed Medline, Embase, Web of Science, and medRxiv databases were searched for all articles containing the keywords “SARS-CoV-2” and “reinfection” published before May 1, 2021.

### Study selection.

The search included all study designs, in humans, reporting paired sequencing of the viral genomes detected during initial and subsequent infection. Reports were included if the sequencing results met at least moderate quality evidence on the basis of CDC investigative criteria or had supporting evidence of reinfection with at least partial sequencing and high clinical suspicion of reinfection.^17^ Reports were excluded if sequencing was not performed as these reports may identify persistent viral shedding rather than true reinfection. Commentaries, correspondence pieces, and reviews that did not include any new data were also excluded. All studies meeting criteria were screened for inclusion based upon title, abstract, and full-text review by J. M. and K. D. All discrepancies were discussed with senior author J. D. K. until consensus was reached.

### Data extraction and items.

Data were extracted from each included report directly into a dedicated excel template. Multiple reports describing different aspects of the clinical course of a single patient were combined into a single entry. The data extracted included country of report, patient’s age, sex, current medications, comorbidities, date of initial positive PCR test, Ct value of initial positive PCR test, degree of symptom burden of initial infection, duration of symptoms of initial infection, date of reinfection positive PCR test, degree of symptom burden of reinfection, duration of symptoms of reinfection, separation between positive PCR tests, viral clade of initial infection and reinfection, notable mutations of either viral genome, and anti-SARS-CoV-2 titers following recovery from initial infection, at presentation of reinfection, and following recovery from reinfection.

### Risk of bias.

Given that all eligible reports included paired genetic sequencing, the data presented here is not a representative sample of all cases of reinfection. However, if less stringent selection criteria were used, then the analysis would be at risk of including many cases that were not true reinfection, which would introduce measurement bias into the analysis.

### Synthesis of results.

Given the heterogeneity of the data available from each case report, it is difficult to generate meaningful summary measures or synthesize results. Rather, common findings among multiple reports were identified and described in this manuscript. Summary statistics were derived, including the median values for continuous variables and percentages for dichotomous variables.

## RESULTS

**Figure 1.**
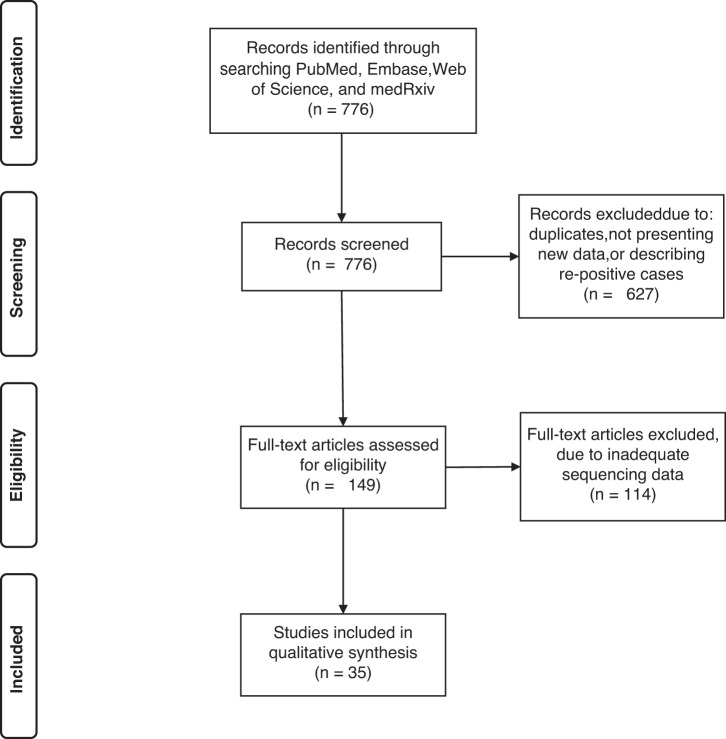
The Preferred Reporting Items for Systematic Reviews and Meta-Analyses (PRISMA) flow diagram detailing the number of screened and included abstracts and articles. In total, 35 articles describing 60 cases met inclusion criteria and were included in the analysis.

The search returned 776 results that were screened to assess whether they met inclusion or exclusion criteria. In total, 35 articles describing the clinical courses of 60 unique individuals were identified that met the criteria.^7^^–^^14^^,^^18^^–^^43^ An additional 114 articles were identified following abstract screening, but were not included in the analysis because they did not have sufficient sequencing results to conclude that the cases were reinfections. In total, 60 cases of verified reinfection were identified in this search. A flow diagram describing the process can be found in Figure 1. Twenty-eight articles were identified in PubMed, two in Embase, one in Web of Science, and three in medRxiv. Four were cohort studies, seven were case series, and 24 were case reports. A detailed breakdown of the relevant parameters reported from each case report can be found in Supplemental Table 1.

Median age of individuals reported to have reinfection was 47 years old (range: 21–92) and 59% (35/59) of individuals were male (the sex of one individual was not reported). Median time to identification of reinfection was 116 days following initial positive test or symptom onset (range: 18–308). About 62% (18/29) of individuals had at least one reported prior medical condition including asthma treated with daily corticosteroids, severe emphysema, hypertension, Waldenstrom’s macroglobulinemia (treated with B cell depleting therapy), allergic rhinitis, type 2 diabetes mellitus, obesity, chronic obstructive pulmonary disease, ischemic heart disease, end-stage renal disease, Paget’s disease, tuberculosis, discoid lupus erythematosus, viral hepatitis, and memory/behavioral disorders.

As a surrogate for viral load (and to rule out false positives) Ct values from PCR testing were reported for 46 cases at initial infection. Median Ct for initial infection was found to be 26.4 (range: 13–36.8). For reinfection, Ct values were reported for 46 cases. Median Ct for reinfection was found to be 24.5 (range: 12–43.3).

During initial infection, 7.5% (4/53) experienced a severe clinical course (requiring intubation or supportive oxygen), 11.3% (6/53) were asymptomatic, and 81.1% (43/53) had a mildly symptomatic clinical course. Upon reinfection, 12.5% (7/56) had a severe clinical course, 25% (14/56) were asymptomatic, and 62.5% (35/56) experienced a mildly symptomatic clinical course. When comparing clinical course, number of symptoms, duration of symptoms, and symptom severity between initial infection and reinfection, 41.5% (22/53) experienced greater symptom burden than initial infection, 17% (9/53) experienced the same symptom burden for both the initial infection and reinfection, and 41.5% (22/53) experienced a milder symptom burden upon reinfection. Among people who were found to have reinfection less than 90 days following initial positive PCR with reported symptom burdens, 80% (12/15) experienced greater symptom burden and 20% (3/15) experienced milder symptom burden. Notably no reported cases of reinfection before 90 days with known lineage were found to be harboring an escape mutation. Among people who were found to have reinfection at least 90 days following initial positive PCR with reported symptom burdens, 24.3% (9/37) experienced greater symptom burden, 24.3% (9/37) experienced the same symptom burden, and 51.4% (19/37) experienced milder symptom burden.

The available phylogenetic information for the cases can be found in Supplemental Table 1. Two cases of reinfection were associated with PANGO lineage B.1.1.7 (WHO Alpha), two with B.1.177, 14 with B.1.160, one with B.1.351 (WHO Beta), one with P.1 (WHO Gamma), and two with P.2 for a total of 47% (22/47) cases with reported lineage for reinfection associated with at least one immune escape mutation. Of note, with regard to immune escape mutants, N440K was reported in two cases, E484K was reported in four cases, and N501Y in four cases. Additionally, 18 of the viral samples at the time of reinfection were identified to have the D614G mutation which has been discussed in literature to be bound with different affinity by neutralizing antibodies formed against the D614 reference sequence although it is not an escape mutant.^44^^,^^45^

Assessment of immunologic response by antibody titer was not routinely performed for all cases. Seroconversion was identified in 72.2% (13/18) of individuals tested following initial infection. At the time of presentation with reinfection, 43.8% (7/16) of individuals were found to have a detectable antibody titer. After recovery from reinfection, 96.3% (26/27) of individuals who were tested for presence of antibodies were found to have seroconverted. These individuals had robust serological responses, identified as long as 3 months following reinfection among individuals in whom follow-up data were available. However, one individual no longer had detectable IgG or IgM titers at day 105 following detection of reinfection.

In terms of infectivity of viral samples from reinfected individuals, viral culture was reported to be attempted in only two cases.^10^^,^^26^ The findings of attempted culture from a diluted nasopharyngeal sample were inconclusive in one case, and negative after two passages in the other case.

## DISCUSSION

### Summary of findings.

Although available data on reinfection cases is somewhat heterogeneous because of the varying clinical protocols used across sites where these cases were identified, some patterns emerge that may warrant further investigation in organized studies of reinfection. This finding also suggests that there may be benefit to developing a standardized international approach to monitoring and reporting reinfection cases for the ease of interpretation given that they appear, at this point, to be relatively rare events. The investigative criteria put forth by the US CDC may serve as a guiding framework for international collaboration in this area as they provide clear criteria and standardized testing recommendations for the workup of potential reinfections.^46^

With regards to viral genomics, a common theme identified in several cases was nonsynonymous mutations in the Spike protein with 45 cases having at least one identified amino acid change. Of particular significance, N440K was reported in two cases, E484K was reported in four cases, and N501Y in four cases, all associated with varying degrees of immune escape. The D614G mutation was also reported in 18 cases. Although this mutation has been shown to result in altered binding affinity of antibodies generated in response to the reference strain, it is not an escape mutant. These findings raise concern for the emergence of an escape mutation that would have a greater potential to reinfect individuals who had previously developed an immune response against a different strain of the virus. This may also be relevant to vaccinated individuals as the emergence of alterations in the spike protein structure as a result of mutations may reduce the effectiveness of vaccines that only target the currently present strains. This is particularly relevant as B.1.1.7, B.1.351, P.1, and P.2 have spread rapidly underscoring the need for adequate surveillance and screening programs among public health entities to identify and characterize emerging and already present variants. This is underscored by early studies indicating that, for strains harboring K417N, E484K, and/or N501Y mutations, there is reduced neutralizing activity of vaccine-elicited monoclonal antibodies.^47^

Of particular interest, the common themes that emerged among reported cases were declining or reduced immune response to initial infection and suspected viral escape mutations. The majority of reported reinfections occurred more than 90 days following initial infection. This corresponds to the time period when neutralizing antibody levels in the blood have been observed to decline and may suggest a potential benefit to periodic booster immunization as more is understood about the durability of immune response. Furthermore, cases of reinfection with greater symptom burden than initial infection tended to occur less than 90 days following initial infection, whereas cases of reinfection after 90 days had a lesser symptom burden than initial infection. Interestingly, this 90-day period aligns with the findings of declining antibody titers found in longitudinal studies of anti-SARS-CoV-2 antibody response following infection and may lend further support to hyperinflammatory state as a primary mechanism of severe illness. It also remains to be seen if a similar finding may be observed among vaccinated individuals as time goes on and antibody titers may begin to decline.

Data was incomplete and heterogeneous for seroconversion. However, among 27 individuals who were tested following reinfection, only one did not have a detectable level of the measured anti-SARS-CoV-2 antibodies. This suggests that the tested individuals were all sufficiently immunocompetent to produce antibodies following initial exposure. Therefore, these cases of reinfection may be indicative of either a decline in circulating antibody level or mucosal immunity below a critical level over time or exposure to new variants of SARS-CoV-2 with sufficient alteration of epitopes to enable reinfection. This may have significant public health implications as this suggests that reinfection is not solely observed in immunocompromised individuals, greatly increasing the size of the potential population at risk.

Alternatively, not all individuals who were tested had detectable antibody titers following initial infection and one individual had received B cell depleting therapy for an unrelated medical condition prior to reinfection. This may suggest that, in these individuals, the immune response from the first exposure was insufficient to prevent reinfection upon subsequent exposure due to either insufficient neutralizing antibody, absent or diminished mucosal immunity, or another potential mechanism.

Serology was not reported for the two individuals who experienced an asymptomatic course of both initial infection and reinfection and thus does not provide any additional information regarding the role of asymptomatic infection on the strength of antibody response or the risk of subsequent infection. However, we did note that all individuals with an asymptomatic initial infection had either mild or another asymptomatic reinfection. Although most of the reinfected individuals in this group had a more severe illness, they did not report moderate or severe illness, suggesting that asymptomatic infection confers a degree of protective immunity lasting beyond the frequently declining antibody response.

Overall, the number of reported cases of reinfection has been low, but prevalence is anticipated to increase in the coming months as more individuals progress beyond the period of initial infection given that it is known reinfection with human coronaviruses often occurs after approximately 12 months.^48^

### Strengths and limitations.

This study attempts to bring together the disparate results of the multiple independent case reports of SARS-CoV-2 reinfection to begin the process of identifying common features that may serve as helpful areas of investigation for future studies. This review was able to identify a sizable number of reinfection cases, which was sufficient to describe several interesting commonalities such as the time to reinfection as well as the symptom burden.

The findings of this review selected for individuals who had sequencing following recovery of initial positive PCR test and were reported in the preprint or published literature. Given our evolving understanding of reinfection and development of standardized approaches, we observed significant amount of heterogeneity in case reports, which can also be attributed to lack of routine sample storage, inconsistent laboratory testing, and the timing of tests such as serology assays being highly variable. Future studies of seroconversion, in particular, are further limited as serology is not routinely performed and cannot be done retroactively once an individual has developed suspected reinfection to assess their status before reexposure. The clinical course of asymptomatic initial infections was likely biased toward symptomatic reinfections because testing strategies are more common among symptomatic individuals.

### Future research questions.

The true prevalence of reinfection and its relative symptom burden remain unclear given that routine repeat testing is not performed for individuals (particularly if asymptomatic) and samples from initial infection are not routinely stored for paired sequencing analysis.

Furthermore, the natural history and causal determinants of reinfection are not well characterized. The timing of reinfection, the degree of infectiousness at the time of reinfection, as well as the dynamics of the immune response triggered by a second exposure have not been clearly elucidated. Similarly, given the variability in testing and the limited number of individuals who had serologic testing prior to presentation with reinfection, the immune response to initial infection in this particular population is not well characterized. Therefore, large population-based surveillance studies and/or more rigorous observational cohorts in which systematic blood sampling occurs before reinfection will be greatly informative of the natural history and casual determinants of reinfection.

It also remains to be seen how relevant these findings may be to understanding the protection from infection conferred by vaccination and whether asymptomatic infection with associated viral shedding may be occurring in that population as well. This would have broad implications on our understanding of the role of vaccination in the effort to reduce the spread and ultimately eradicate this condition.

## CONCLUSION

In total, 60 reported cases were identified that met inclusion criteria through our search strategy, emphasizing that although there may be a bottleneck in capturing and reporting these data, reinfection continues to be a relatively rare event after more than a year since the pandemic began. We identified potential reinfection clinical phenotypes based on the timing of reinfection (before or after 90 days since initial positive PCR test), similar Ct values across illnesses, presence of variants of concern (VOC) and non-VOCs, and nearly uniform seroconversion after 28 days from reinfection. These findings suggest that reinfection occurs across a wide population demographic. There continues to be a need for a structured investigation of reinfection cases to determine natural history and causal determinants of reinfection on a population level. In particular, a study assessing the dynamics of anti-SARS-CoV-2 titers at three primary time points (following recovery from initial infection, at presentation of reinfection, and following recovery from reinfection), viral genetic sequences of initial and reinfection strains, symptom burden, and comorbid conditions of individuals experiencing reinfection would help to elucidate some of the questions that emerge from this analysis. An essential feature of such a study would be a detailed assessment of time points prior to reinfection. This underscores the need for large-scale active surveillance programs to be able to capture these data points. These findings may also offer some insight into planning for long-term investigation of vaccine efficacy and the risk of subsequent infection as well as the potential need for booster immunization on a regular basis.

## Supplemental Table


Supplemental materials
